# Sulforaphane Reduces the Chronic Inflammatory Immune Response of Human Dendritic Cells

**DOI:** 10.3390/nu15153405

**Published:** 2023-07-31

**Authors:** Laura Fernandez-Prades, Mariano Brasal-Prieto, Gonzalo Alba, Victoria Martin, Sergio Montserrat-de la Paz, Marta Cejudo-Guillen, Consuelo Santa-Maria, Hala Dakhaoui, Beatriz Granados, Francisco Sobrino, Francisca Palomares, Soledad Lopez-Enriquez

**Affiliations:** 1 Department of Medical Biochemistry and Molecular Biology, and Immunology, School of Medicine, University of Seville, Av. Sanchez Pizjuan s/n, 41009 Seville, Spain; laurafprades@gmail.com (L.F.-P.); marbrapri123@gmail.com (M.B.-P.); galbaj@us.es (G.A.); vicmarsan4@alum.us.es (V.M.); delapaz@us.es (S.M.-d.l.P.); hala.bouita@hotmail.com (H.D.); fsobrino@us.es (F.S.); 2 Department of Pharmacology, Pediatry, and Radiology, School of Medicine, University of Seville, Av. Sanchez Pizjuan s/n, 41009 Seville, Spain; mcejudo@us.es; 3 Department of Biochemistry and Molecular Biology, School of Pharmacy, University of Seville, 41012 Seville, Spain; csm@us.es; 4 Distrito Sanitario Málaga, Servicio Andaluz de Salud, 29006 Málaga, Spain; beitagranados@hotmail.com

**Keywords:** nutraceutical, dendritic cells, T lymphocytes, anti-inflammatory response, autophagy, apoptosis

## Abstract

Background: Sulforaphane (SFN) is an isothiocyanate of vegetable origin with potent antioxidant and immunomodulatory properties. The characterization of its pleiotropic activity in human dendritic cells (DCs) is poorly summarized. The aim of this work was to study the immunomodulatory power of SFN in response to an inflammatory microenvironment on human monocyte-derived DCs (moDCs). Methods: We studied the immunological response induced by SFN. Apoptosis and autophagy assays were performed using flow cytometry on moDCs and a cancer cell line (THP-1). These included moDC maturation, lymphocyte proliferation and cytokine production under different experimental conditions. We investigated whether these results were associated with an inflammatory microenvironment induced by lipopolysaccharides (LPSs). Results: Our results demonstrated that SFN could interact with moDCs, significantly reducing the autophagy process and enhancing apoptosis similarly to cancer cell line THP-1 cells in a chronic inflammatory microenvironment. Under chronic inflammation, SFN modulated the phenotypical characteristics of moDCs, reducing the expression of all markers (CD80, CD83, CD86, HLA-DR and PD-L1). SFN significantly reduced the Th2 proliferative response, with a decrease in the IL-9 and IL-13 levels. Although we did not observe any changes in the regulatory proliferative response, we noted an increase in the IL-10 levels. Conclusions: These findings demonstrate that SFN exerts protective effects against LPS-induced inflammation via the modulation of moDCs/T cells towards a regulatory profile. SFN may be a potential candidate for the treatment of pathologies with an inflammatory profile.

## 1. Introduction

Sulforaphane (SFN), an isothiocyanate of plant origin found in cruciferous vegetables such as broccoli, presents potent antioxidant [1] and immunomodulatory properties, including anti-inflammatory, anticarcinogenic (including a histone deacetylase inhibitory action), antimicrobial, anti-Alzheimer’s and anti-diabetes effects [1,2,3,4]. These pleiotropic activities are derived from its ability to influence multiple signaling pathways, highlighting the nuclear factor erythroid 2-related factor 2 (Nrf2) and heme oxygenase 1 (HO-1) pathways [1,5] in different immune cells such as monocytes, macrophages, lymphocytes and dendritic cells (DCs) [6]. These pathways have also been studied in microglia and neurons [5,7,8]. In this sense, SFN exerts an anti-neuroinflammatory effect on microglia through c-Jun N-terminal kinase (JNK)/activator protein 1 (AP-1)/NF-κB pathway inhibition and Nrf2/HO-1 pathway activation [5]. It has been demonstrated that the SFN activation of this signaling Nrf2 pathway in DCs produces polarization towards a tolerogenic phenotype [9,10,11,12]. SFN Nrf2 activation restored the age-related decline of the Th1 response in DCs in an animal model [10]. 

DCs are professional antigen-presenting cells (APC). They are essential for interactions with naive T lymphocytes [13]. Through a maturation process, DCs can modulate the tolerant or effector immune responses mediated by different subtypes of T lymphocytes and microenvironments [14,15,16,17], leading to a more effective immune response. Wang et al. demonstrated that, in humans, SFN could promote T-cell activation by DCs through the modulation of regulatory molecules. They proposed that this nutraceutical could be used in the cotreatment of cancer [11]. Qu et al. demonstrated that SFN could epigenetically regulate inflammation in LPS-induced porcine moDCs [18]. In contrast, the SFN treatment of DCs inhibited IL-12 and IL-23 production, severely suppressing the Th1 and Th17 development of T cells primed with SFN-treated DCs [9]. The interaction of SFN with DCs reduces inflammation, preventing tissue damage and contributing to a balanced immune environment. Advancing the studies of SFN and DCs enables in-depth investigations into how this nutraceutical can modulate the immune function and immune response, thus allowing the development of new therapies and immunomodulatory approaches for various diseases such as cancer or allergies.

We previously demonstrated that DCs have the capacity to induce the proliferation of specific lymphocytes from drug- or food-allergic patients compared to other APCs such as B cells or monocytes, improving the in vitro diagnosis of these patients [14,15,16]. Our group has also presented data for the characterization of pathway signaling in DCs or neutrophils [19,20,21,22]. Little is known about the initial steps mediated by the innate immune system after the uptake of SFN nutraceuticals and the T-lymphocyte-mediated response generated in a proinflammatory environment. 

In this study, we demonstrate the novel finding that SFN in an inflammatory environment can potentially reduce the type 2 immune response in human DCs. We studied the proliferation and modulation of the T-cell-mediated response under experimental conditions. Our results indicated that SFN in a proinflammatory environment exerts an important effect on the phenotypic changes of DCs, inducing T-cell activation, reducing the Th2 proliferative response and increasing the IL-10 levels in an in vitro situation with LPS.

## 2. Materials and Methods

### 2.1. Subjects of Study

The biological samples used for this work were obtained from twelve healthy subjects older than 18 years. The sample collection was approved by the Research Ethics Committee of the Hospitales Universitarios Virgen Macarena Virgen del Rocío (code: ID-SOL2022-21799). Prior to the sample collection, each subject signed an informed consent for inclusion in the study.

### 2.2. Sample Collection and Cell Culture

The samples from the controls were processed following the current procedures immediately after their reception. These were kindly provided by BBSSPA, unit HH.UU, Virgen Macarena Virgen del Rocío. PBMCs and moDCs were obtained from peripheral blood (40 mL) from the healthy subjects included in the study. PBMCs were isolated by centrifugation on a gradient using Ficoll (Sigma, Madrid, Spain) with standardized protocols. Monocytes were purified from the PBMCs by a positive selection with anti-CD14 magnetic microbeads following the manufacturer’s protocol (Miltenyi Biotec, Bergisch Gladbach, Germany). These had a purity greater than 95%, as assessed by flow cytometry CD14^−^ cell fractions were frozen for further use. Monocytes (CD14^+^ cells) were cultured in a complete medium as previously described by us for the generation of monocyte-derived DCs [19].

### 2.3. Generation of DCs

Monocyte-derived DCs (moDCs) were generated from CD14^+^ monocytes as described in [23] by culturing them in a complete medium with the addition of 200 ng/mL GM-CSF and 100 ng/mL IL-4 (both from R&D Systems Inc, Minneapolis, MN, USA) maintained at 5% CO_2_ and 37 °C, as described in [19].

### 2.4. Cytotoxicity Assays

MoDC and THP-1 cells seeded in 96-well plates (5 × 10^4^ cells/well) were incubated in the presence or absence of 100 ng/mL LPS of Escherichia coli 0127:B8 (Sigma-Aldrich, St. Louis, MO, USA) as a positive control of chronic inflammation and 10, 20 and 30 μM of SFN (Sigma-Aldrich), the nutraceutical of the study, for 48 h. THP-1 was included as a model of a cancer cell line, because it consisted of monocytes isolated from peripheral blood from an acute monocytic leukemia patient. After the exposure time, the effect on cell growth/viability was analyzed by flow cytometry using a LIVE/DEAD™ viability kit (Thermo Fisher Scientific, Waltham, MA, USA). Cell survival was measured as the percentage of live cells (viability%) compared with non-treated control cells.

### 2.5. Activation Assays of DCs

On day 5, moDCs and THP-1 were seeded in 96-well plates (5 × 10^4^ cells/well) and treated with 10 µM of SFN for 48 h. This was the optimal concentration for the maturation of moDCs. It proved to have noncytotoxic effects in the presence or absence of 100 ng/mL of LPS added 1 h before adding 10 µM of SFN, inducing a strong inflammatory response as a model of chronic inflammatory disease [24].

### 2.6. Apoptosis Assays

To detect the type of cell death that SFN could trigger, an FITC annexin V apoptosis detection kit (BD Biosciences Pharmingen) was used following the manufacturer’s instructions. Briefly, moDC and THP-1 cells were seeded in 96-well plates (5 × 10^4^ cells/well) and treated with 10 µM of SFN and/or LPS for 48 h. The samples were then incubated at room temperature in the dark for 15 min with FITC annexin V and propidium iodide (PI). Cells were acquired by MACSQuant VYB flow cytometry (Miltenyi Biotec BV & Co KG, Bergisch Gladbach, Germany), and the data were analyzed using FlowJo software (Tree Star, Inc., Ashland, OR, USA). The results were expressed as the percentages of positive cells. 

### 2.7. Autophagy Assays

Treated or non-treated moDCs (5 × 10^4^ cells/well) were collected for the detection of autophagy after 48 h of culture at 37 °C and 5% CO_2_. Cells were washed and fixed in 1% paraformaldehyde (PFA) for 10 min at room temperature (RT) using gentle shaking. The cells were then permeabilized with a rinse buffer (PBS; 20 mM tris-HCl, 0.15 M NaCl and 0.05% Tween-20) and incubated at RT with gentle shaking for 30 min. The cells were washed with a blocking solution (PBS and 3% BSA) and then incubated with an LC3B-APC antibody (Novus Biologicals, Centennial, CO, USA) for 30 min in the dark at RT. Finally, the cells were acquired by flow cytometry and analyzed using FlowJo software, as previously described. The fold change was calculated for LC3B (%LC3B expression on stimulated moDCs/% LC3B expression on unstimulated moDCs).

### 2.8. Phenotypic Analysis of moDCs

Treated or non-treated moDCs (5 × 10^4^ cells/well) were incubated for 15 min at RT with specific mAbs (HLA-DR–FITC, PD-L1-PE, CD86-PerCP, CD80-PE-Cy7 and CD83-APC (Biolegend, San Diego, CA, USA) or with an isotype-matched control). The cells were acquired by flow cytometry and analyzed using FlowJo software, as previously described. The results were expressed as the fold change of the percentage of expression for each surface marker on the moDCs. The fold change was calculated for each marker (% marker expression on stimulated moDCs/% marker expression on unstimulated moDCs).

### 2.9. Specific Proliferative Response 

The specific proliferation of different lymphocyte subpopulations was evaluated using, as APCs, autologous moDCs pre-stimulated with 10 µM of SFN in the presence or absence of LPS for 48 h, as previously described. Proliferation was determined using a 5,6-carboxyfluorescein diacetate N-succinimidyl ester (CFSE) dilution assay (Thermo Fisher Scientific). A total of 1.5 × 10^6^/mL prelabeled CD14^-^ cells were cultured with moDCs pre-stimulated with different experimental combinations (10:1 ratio) at a final volume of 250 μL of the complete medium in 96-well plates for 6 days at 37 °C and 5% CO_2_. Unstimulated moDCs were used as a negative proliferative control, and 10 μg/mL of phytohemagglutinin (Sigma-Aldrich) was used as a positive proliferative control. The proliferative responses were assessed by flow cytometry, analyzing the CFSE^low^ expression in the different cell subsets as T lymphocytes (CD3^+^CD4^+^T-, CD3^+^CD4^+^CRTH2^+^Th2-, CD3^+^CD4^+^CRTH2^-^Th9- and CD3^+^CD4^+^CD25^+^CD127^-^ as Treg cells). Further details of the fluorescent antibodies used are presented in Table S1 in the Supplementary Materials. The results were expressed as the fold change of the percentage of CFSE^low^ for each cell subpopulation. The fold change was calculated for each cell subset (% CFSE^low^-stimulated moDCs:CD14^-^ PBMCs/% CFSE^low^ unstimulated moDCs:CD14^-^ PBMCs).

### 2.10. Cytokine Production

Cytokine production (IL-4, IL-5, IL-9, IL-10, IL-13 and IFN-γ) from cell supernatant cultures (moDCs:CD14^−^ PBMCs) were collected (100 µL) after 7 days. The cytokine production was determined using a human ProcartaPlex Multiplex Immunoassays kit (Thermo Fisher Scientific) following the manufacturer’s instructions. Briefly, the samples (50 µL) and standards were incubated (overnight at 4 °C) with the magnetic bead mix in a 96-well plate. After that, the biotinylated antibody was added and incubated for 300 min at RT, and following that, streptavidin was added and incubated for 300 min at RT. After each incubation, washing steps were performed. Finally, the plate was prepared with a reading buffer to be detected using Bio-Plex 200 (Bio-Rad, Hercules, CA, USA) [16,25]. Data were analyzed using Bio-Plex Data Analysis Software (Bio-Rad Bio-Rad Laboratories, Inc, Hercules, CA, USA). The results were expressed as the fold change for the concentration (pg/mL) of each cytokine (cytokine production from supernatant of stimulated cell cocultures/cytokine production from supernatant of unstimulated cell cocultures).

### 2.11. Statistical Analysis

The data were analyzed using the Shapiro−Wilk test to determine the normal distribution. Most variables were fitted to a nonparametric distribution. The Friedman test was used to discover significant differences due to the effects of different LPS, SFN and SFN+LPS treatments between subjects from the same group. If the Friedman test indicated the existence of significant differences between treatments, we used the Wilcoxon signed rank test to compare pairs of related samples. This resulted in three post hoc tests (LPS vs. SFN, LPS vs. SFN plus LPS and SFN vs. SFN plus LPS). The significant differences were reported as * and **, representing *p*-values < 0.05 and < 0.01, respectively. The statistical analysis was performed using GraphPad Prism7.

## 3. Results and Discussion 

### 3.1. Sulforaphane Induces Apoptosis in Inflammatory moDCs

The SFN optimal concentrations used on moDCs from healthy subjects were analyzed by a dose–response curve assay. After 48 h in the culture, we measured the percentage of viable moDCs following exposure to increasing doses of SFN (10, 20 and 30 μM) (Figure S1A). We assessed the viable treated moDCs by binding a fluorescent dye to free amines inside the cell membrane of the necrotic bodies through the flow cytometer. When we compared the moDCs without stimulation or stimulated with increasing doses of SFN, we observed that the cell viability was strongly engaged from 20 μM of SFN (Figure S1A). This was in line with data from Wang et al. [11], although they noted that their inhibition of the cell viability began at 25 μM. We selected 10 μM of SFN as the optimal in vitro assay after exposure to an increasing dose, because at this concentration, SFN did not affect the viability of the moDCs (Figure S1B). Similarly, when we added 100 ng/mL of LPS as a positive control of chronic inflammation, the viability of the moDCs was not affected. When we added 10 μM of SFN after inducing moDC chronic inflammation by adding 100 ng/mL of LPS, we observed a significant decrease in the viability of the moDCs with respect to both the negative and positive controls (Figure S1B). We hypothesized that, under chronic inflammation, SFN could act on affected moDCs, thereby promoting apoptosis. This was in line with other authors, who proposed that the pharmacological induction of apoptosis could lead to entirely new therapies for chronic inflammatory diseases [26,27]. Carstensen et al. demonstrated that exposure to an inflammatory environment maintained over time induced apoptosis in DCs [28]. In our study, this action could be enhanced by an anti-inflammatory agent such as SFN.

**Figure 1:**
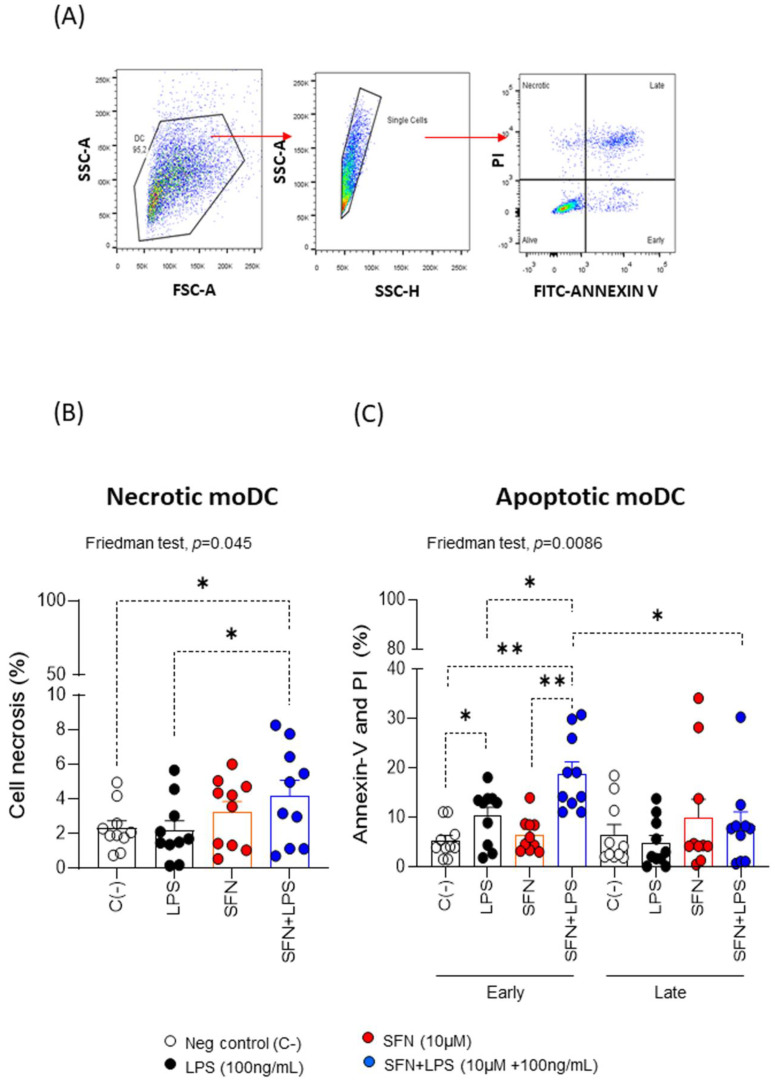
(**A**) Gate strategy followed for a flow cytometry analysis showing necrotic and apoptotic moDCs. (**B**,**C**) Necrosis and apoptotic percentages of moDCs (N = 10) under different experimental conditions. PI: propidium iodide; LPS: lipopolysaccharide (100 ng/mL); SFN: sulforaphane (10 µM). The bars with symbols represent the means and standard errors of the percentage of viability, necrosis and apoptosis on moDCs. The Friedman test was used to detect differences in related samples across multiple comparisons, representing significant *p*-values. The Wilcoxon test was used for pairwise comparisons of related samples, representing significant *p*-values. * *p* < 0.05; ** *p* < 0.01.

To further characterize the type of cell death that SFN could be triggered on moDCs and LPS-stimulated moDCs, we measured the apoptosis (Figure 1A). The results revealed that SFN alone induced cell apoptosis with increasing doses for both early and late apoptosis. The analysis of the SFN effect in an inflammatory microenvironment induced by LPS indicated that SFN induced significant changes in necrosis and apoptosis on moDCs (Figure 1B,C). This correlated with studies that demonstrated that, in cancer cells with an inflammatory profile, SFN could induce apoptosis [29]. To confirm these results, we studied the effect of SFN on THP-1 cells in non-inflammatory and inflammatory microenvironments with LPS. The data showed that SFN, under this inflammatory microenvironment, induced necrosis and apoptosis on the cancer cell line (Figure S2). Our findings suggest that SFN-induced apoptosis in an inflammatory microenvironment may be a promising strategy for cancer control.

### 3.2. Sulforaphane Decreases Autophagy in Inflammatory moDCs

**Figure 2:**
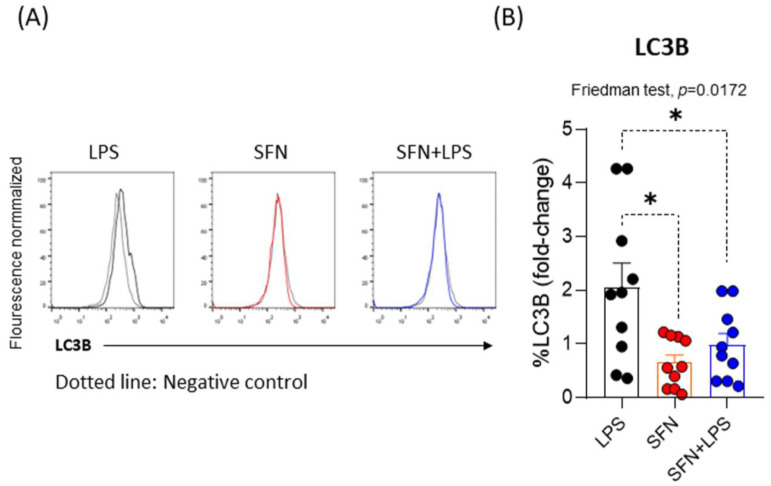
(**A**) Histograms representing the mean normalized fluorescence intensity of LC3B under different experimental conditions. (**B**) Fold change of LC3B on moDCs (N = 10) under different experimental conditions. LPS: lipopolysaccharide (100 ng/mL); SFN: sulforaphane (10 µM). The bars with symbols represent the means and standard errors of the fold changes. The Friedman test was used to detect differences in related samples across multiple comparisons, representing significant *p*-values. The Wilcoxon test was used for pairwise comparisons of related samples, representing significant *p*-values. * *p* < 0.05.

Autophagy is involved in the tolerogenic and immunogenic functions of DCs, depending on the microenvironment [30]. Increased autophagy has been detected in macrophages and DCs upon virus infections [31,32]. Blanchet et al. observed that HIV-1 inhibited autophagy in DCs, but LPS increased it [33]. LPS-treated DCs under hypoxia increased autophagy, promoting the survival of DCs [34]. Our results demonstrated that LPS induced increased autophagy in moDCs subjected to a chronic inflammatory environment. This was in line with previous studies [33], but SFN (alone) did not alter autophagy. SFN significantly decreased LPS-induced autophagy (Figure 2). Knowledge about the involvement of autophagy in DCs has increased [30], but understanding the functioning of autophagy in tolerogenic or immunogenic DCs and its interconnection with apoptosis require a deeper comprehension.

Considering all the results, it is tempting to speculate that, in an inflammatory profile (such as those occurring in cancer pathologies), there is an inhibition of autophagy, enhancing SFN-induced apoptosis [29,35]. These findings provide a premise for the use of nutraceutical agents as a treatment for inflammatory diseases.

### 3.3. Sulforaphane Induces Phenotypic Changes in Inflammatory moDCs 

MoDCs could be activated by an inflammatory microenvironment induced by LPS. This led to changes in the expression of different costimulatory markers (CD80, CD86, CD83, HLA-DR and PD-L1), which promoted the immune response and recruited other types of immune cells to the site of inflammation, as with T lymphocytes [36]. An evaluation of LPS-induced maturational phenotypic changes in the expression of the cell surface markers of activation/regulation (CD83 and PD-L1), maturation (CD80 and CD86) and antigen presentation (HLA-DR) indicated that there was a significant increase in all markers compared with SFN. 

**Figure 3:**
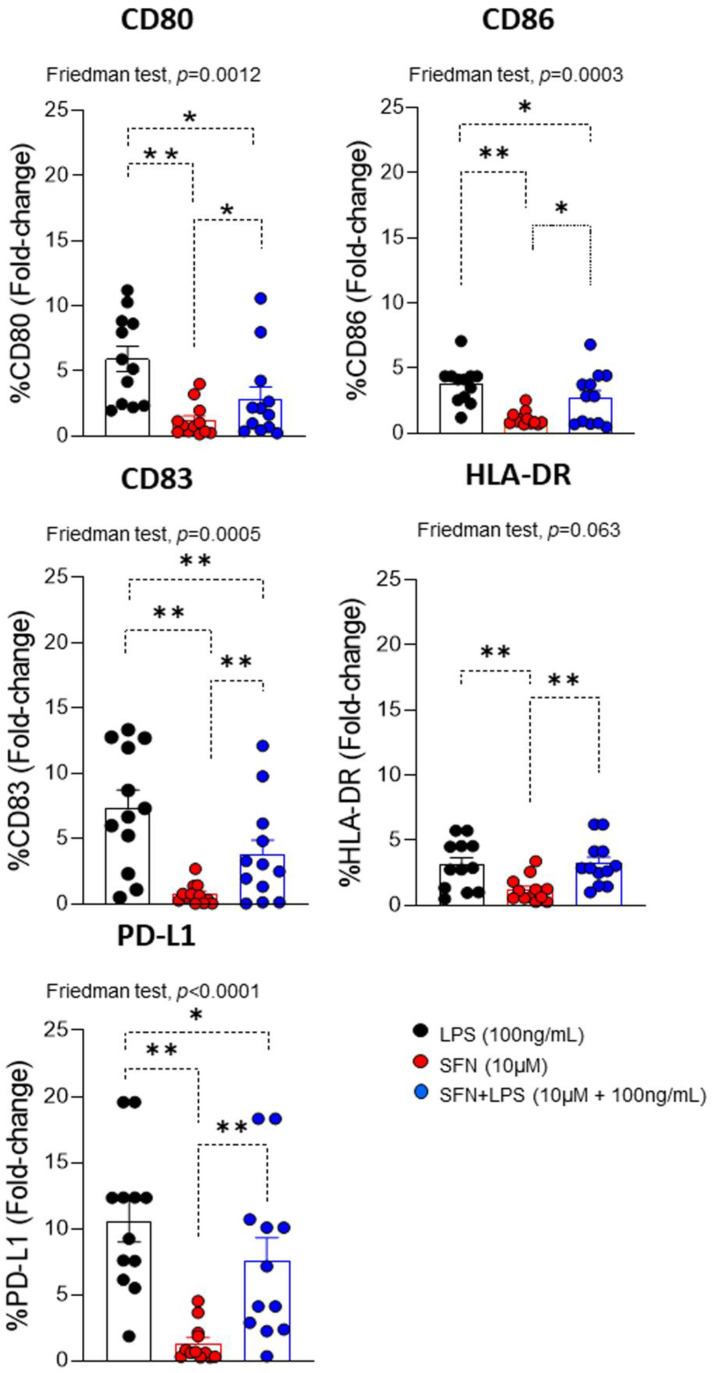
Fold changes of CD80, CD83, CD86, HLA-DR and PD-L1 on moDCs (N = 12) under different experimental conditions. LPS: lipopolysaccharide (100 ng/mL); SFN: sulforaphane (10 µM). The bars with symbols represent the means and standard errors. The Friedman test was used to detect differences in related samples across multiple comparisons, representing significant *p*-values. The Wilcoxon test was used for pairwise comparisons of related samples, representing significant *p*-values. * *p* < 0.05; ** *p* < 0.01.

SFN-stimulated moDCs downregulated these markers (Figure 3). This effect agreed with previous studies in which SFN was shown to inhibit the differentiation of immature to mature moDCs through the downregulation of CD40, CD80 and CD86 expressions [18]. An analysis of the effect of SFN in an inflammatory microenvironment induced by LPS indicated that SFN downregulated the expression of CD80, CD83, CD86 and PD-L1 compared to LPS (alone) (Figure 3). It has recently been noted that LPS stimulation increases PD-L1 expression in cancer cells [37]. 

Several recent studies have comprehensively discussed the role of the immune system in the central nervous system (CNS). Microglia play essential roles in monitoring and performing rapid responses to changes in the CNS and constitute the main resident immune cell population of the brain [38]. Given that monocytes originate from hematopoietic stem cells, whereas microglia are descendants of yolk sac erythro-myeloid progenitors, under inflammatory pathological conditions, monocytes may infiltrate the brain as monocyte-derived macrophages and moDCs and acquire a microglia-like phenotype [39]. Taken together, our results in moDCs lead us to speculate that SFN could improve the neuroinflammation condition, suggesting that monocytes infiltrating the brain could differentiate into in cells like microglia but with similar characteristics and functions as moDCs (under an inflammatory microenvironment induced by LPS). In this sense, the phenotype of these cells would be modified by SFN and therefore regulate the immune response in the CNS. In fact, it has been described that PD-L1 expression on microglia exerts self-protection from inflammation [40]. 

Our results indicated that SFN in an inflammatory microenvironment could modulate the PD-L1 pathway and could provide an antitumor effect. Taken with the current data, this suggests that SFN has a protective role in the presence of LPS. SFN has been demonstrated to inhibit the LPS-stimulated inflammatory response in human monocytes [41]. This capacity to modulate the moDC phenotype in an inflammatory microenvironment has also been described for other nutraceuticals such as apigenin [42]. 

### 3.4. Sulforaphane Reduces the Th2 Proliferative Response under an Inflammatory Microenvironment

Our findings indicated that SFN in an inflammatory environment could modulate the activation and maturation of moDCs, suggesting that they may be involved in the next steps of the immune response where moDCs interact with T lymphocytes. To examine this possibility, we determined the proliferative response using pre-stimulated homologous moDCs under different experimental conditions. For this, different subpopulations of T lymphocytes (CD3^+^CD4^+^T-, CRTH2^+^Th2-, CRTH2^-^Th9- and Treg cells) were assayed.

**Figure 4:**
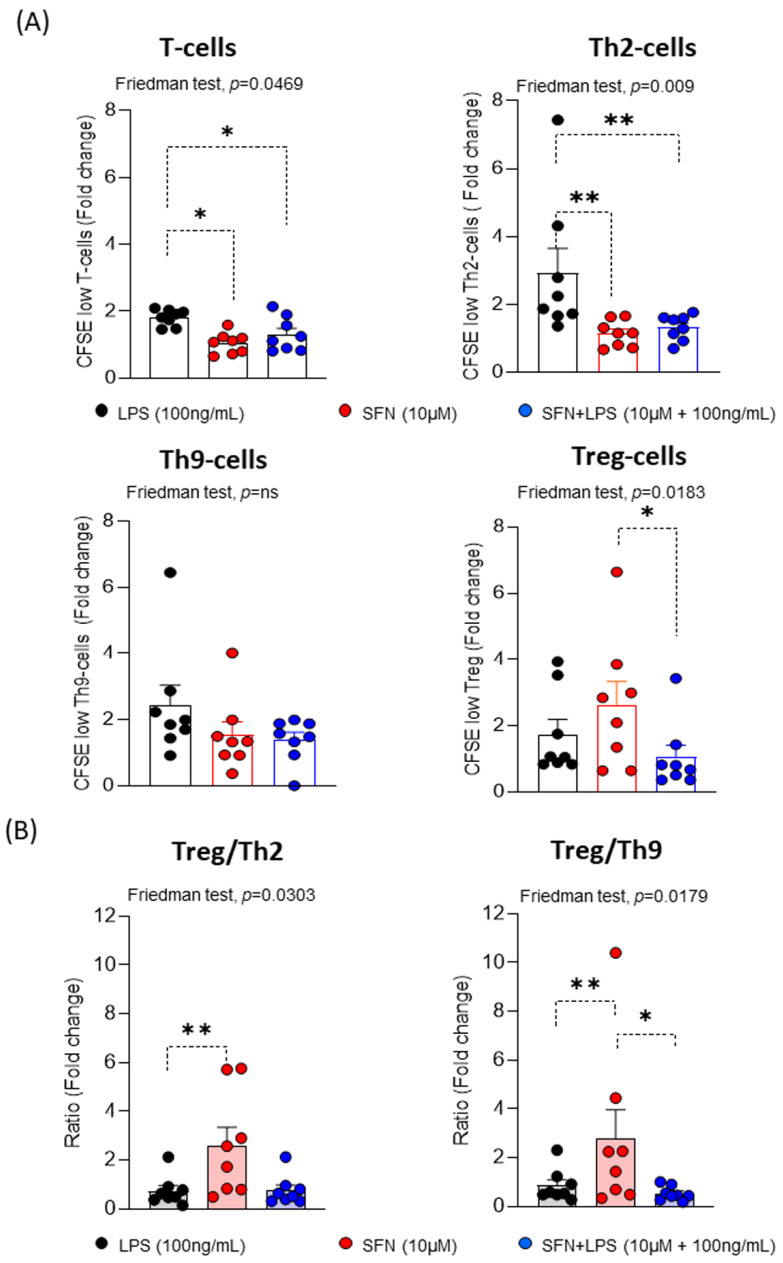
(**A**) Fold changes for the percentages of CFSE^low^ T, Th2, Th9 and Treg cells (N = 8), and (**B**) ratios of the fold changes of Treg/Th2 and Treg/Th9 under different experimental conditions. LPS: lipopolysaccharide (100 ng/mL); SFN: sulforaphane (10 µM). The bars with symbols represent the means and standard errors. CFSE; 5,6-carboxyfluorescein diacetate N-succinimidyl ester. The Friedman test was used to detect differences in related samples across multiple comparisons, representing significant *p*-values. The Wilcoxon test was used for pairwise comparisons of related samples, representing significant *p*-values. * *p* < 0.05; ** *p* < 0.01.

Our results indicated that SFN, regardless of the inflammatory microenvironment, led to significantly decreased CD3^+^CD4^+^T- and CRTH2^+^Th2- cell proliferation compared to LPS. There were no changes observed in the proliferative responses of the CRTH2-Th9- cells (Figure 4). These results suggest that SFN in an inflammatory microenvironment induces a reduction in the type 2 immune response. It has previously been described that SFN suppressed the levels of GATA3 and IL-4 expression in an asthma animal model, demonstrating the regulation of Th2 immune responses [43]. The proliferative response of Treg cells significantly increased under SFN stimulation compared to SFN+LPS. The ratios of the Treg/Th2 cells and Treg/Th9 cells were significantly higher in the presence of SFN than SFN+LPS, suggesting that SFN displays anti-inflammatory features and induces a Treg balance, as has been described for other nutraceuticals [44,45].

**Figure 5:**
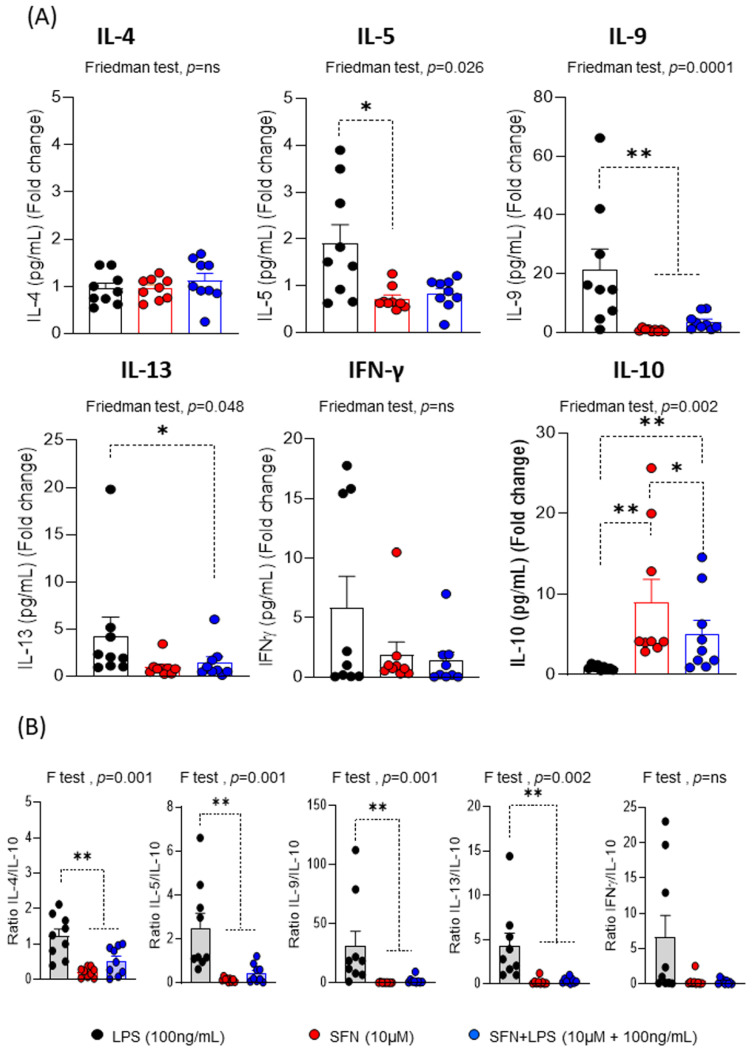
(**A**) Fold changes of the concentration for each cytokine (N = 9). (**B**) Ratios of the fold changes of IL-4/-, IL-5/-, IL-9/-, IL-13/- and IFN-γ/IL-10 under different experimental conditions. LPS: lipopolysaccharide (100 ng/mL); SFN: sulforaphane (10 µM). The bars with symbols represent the means and standard errors. CFSE: 5,6-carboxyfluorescein diacetate N-succinimidyl ester. The Friedman (F) test was used to detect differences in related samples across multiple comparisons, representing significant *p*-values. The Wilcoxon test was used for pairwise comparisons of related samples, representing significant *p*-values. * *p* < 0.05; ** *p* < 0.01.

Regarding the cytokine profile produced during the proliferative response, the most interesting results indicated that SFN in the inflammatory microenvironment induced a decrease in the levels of IL-5, IL-9, IL-13 and IFN-γ compared to LPS, but this was only significant for IL-9 and IL-13 (Figure 5A). The changes that were observed in the levels of the inflammatory and proinflammatory cytokines were consistent with the ability of SFN to suppress type 2 cytokines in an asthma animal model [43].

We previously demonstrated that SFN inhibits the IFN-γ levels [46], as has been demonstrated by other natural substances with anti-inflammatory and/or immunomodulatory activities such as curcumin [47] or sanguinarine [48]. Our results indicated that SFN has a potent effect on the inflammatory and proinflammatory patterns induced by T cells. After 1 h of LPS stimulation, SFN significantly increased IL-10 production compared to LPS (Figure 5A). The IL-4/IL-10, IL-5/IL-10, IL-9/IL-10 and IL-13/IL-10 ratios were significantly lower in the presence of SFN than LPS, suggesting that SFN displayed an anti-inflammatory feature (Figure 5B). SFN stimulated a stable regulatory response under inflammatory conditions, as well as a low production of inflammatory cytokines. Supporting these data, SFN attenuated intestinal inflammation by increasing the IL-10 levels in an animal model [49]. 

These data suggest that SFN in inflammatory conditions may change inflammatory cytokine production. Under chronic inflammation, SFN could modulate the moDC phenotype to interact with T cells and generate a specific proliferative response with a regulatory immune pattern and a decrease in Th2 effector cells. SFN regulated this cell interaction (moDCs/T cells) to induce a regulatory response under inflammatory conditions. 

## 4. Conclusions

In this study, we demonstrated that SFN exerts protective effects against LPS-induced inflammation via the modulation of moDCs/T cells. SFN interacted with moDCs and could reduce autophagy and increase apoptosis in a chronic inflammatory microenvironment, as has been described in cancer. Under these conditions, SFN changed the phenotypic markers of moDCs and modulated the Th2 proliferative response with a reduction in anti-inflammatory cytokines and an increase in the profile of regulatory cytokines. SFN may be a potential candidate for use in the treatment of pathologies with an inflammatory profile such as inflammatory diseases—specifically, inflammatory bowel diseases—alone or in combination with a base treatment. A future research direction could be to explore the use of SFN in allergic diseases with a Th2 immune profile, such as food allergies or neurodegenerative diseases. Further studies are necessary to understand the mechanisms underlying the beneficial effects of SFN on the inflammatory process. 

## Data Availability

No new data were created or analyzed in this study. Data sharing is not applicable to this article.

## Supplementary Materials

The following supporting information can be downloaded at: https://www.mdpi.com/article/10.3390/nu15153405/s1, Table S1: Monoclonal antibodies and probes used for flow cytometry. Figure S1. (A) Viability of moDCs treated with different concentrations of SFN after 48 h. Each symbol represents different SFN concentrations. (B) Cell viability percentages on moDCs under different experimental conditions. LPS: lipopolysaccharide (100 ng/mL); SFN: sulforaphane (10 µM). The bars with different symbols represent the mean and standard error of percentages of viability, necrosis and apoptosis on THP-1-cells. The Friedman test was used to detect differences in related samples across multiple comparisons, representing significant *p*-values. The Wilcoxon test was used for pairwise comparisons of related samples, representing significant *p*-values as * (*p* < 0.05) and ** (*p* < 0.01). Figure S2. (Up) Viability and necrosis percentage on THP-1-cells. (Down) Apoptotic percentages of THP-1-cells (N = 5) under different experimental conditions. LPS: lipopolysaccharide (100 ng/mL); SFN: sulforaphane (10 µM). The bars with symbols represent the means and standard errors of percentage of viability, necrosis and apoptosis on THP-1-cells. The Friedman test was used to detect differences in related samples across multiple comparisons, representing significant *p*-values. The Wilcoxon test for pairwise comparisons in related samples, representing significant *p*-values as * (*p* < 0.05) and ** (*p* < 0.01).

## Abbreviations

APC	Antigen-presenting cells
CFSE	Carboxyfluorescein diacetate N-succinimidyl ester
DCs	Dendritic cells
HO-1	Heme oxygenase 1
LPS	Lipopolysaccharide
moDCs	Monocyte-derived DCs
Nrf2	Nuclear factor erythroid 2-related factor 2
SFN	Sulforaphane
RT	Room temperature
