# Supplementary material for: Sulforaphane Reduces the Chronic Inflammatory Immune Response of Human Dendritic Cells
Source: Nutrients. 2023 Jul 31;15(15):3405. doi: 10.3390/nu15153405 (PMC10421388; doi:10.3390/nu15153405)
Supplement: Supplementary file 1 [file nutrients-15-03405-s001.zip › nutrients-2461316-supplementary.pdf]

Supplementary Materials

Sulforaphane Reduces the Chronic Inflammatory Immune Response of Human Dendritic Cells

Laura Fernandez-Prades [1,†], Mariano Brasal-Prieto [1,†], Gonzalo Alba [1], Victoria Martin [1], Sergio Montserrat-de la Paz [1], Marta Cejudo-Guillen [2], Consuelo Santa-Maria [3], Hala Dakhaoui [1], Beatriz Granados [4], Francisco Sobrino [1], Francisca Palomares [1,*,‡] and Soledad Lopez-Enriquez [1,*,‡]

[1] Department of Medical Biochemistry and Molecular Biology, and Immunology, School of Medicine, University of Seville, Av. Sanchez Pizjuan s/n, 41009 Seville, Spain
[2] Department of Pharmacology, Pediatry, and Radiology, School of Medicine, University of Seville, Av. Sanchez Pizjuan s/n, 41009 Seville, Spain
[3] Department of Biochemistry and Molecular Biology, School of Pharmacy, University of Seville, 41012 Seville, Spain
[4] Distrito Sanitario Málaga, Servicio Andaluz de Salud, 29006 Málaga, Spain

Table S1: Monoclonal antibodies and probes used for flow cytometry.

MoAbs	Fluorochrome	Clone	Vendor
CD3	Pacific Blue	UCHT1	BioLegend
CD4	APC	SK3	BD
CD25	PECy7	M-A251	BD
CD127	PE	HIL-7R-M21	BD
CRTH2	PE	REA598	Milteny Biotec

MoAb: Monoclonal Antibody.

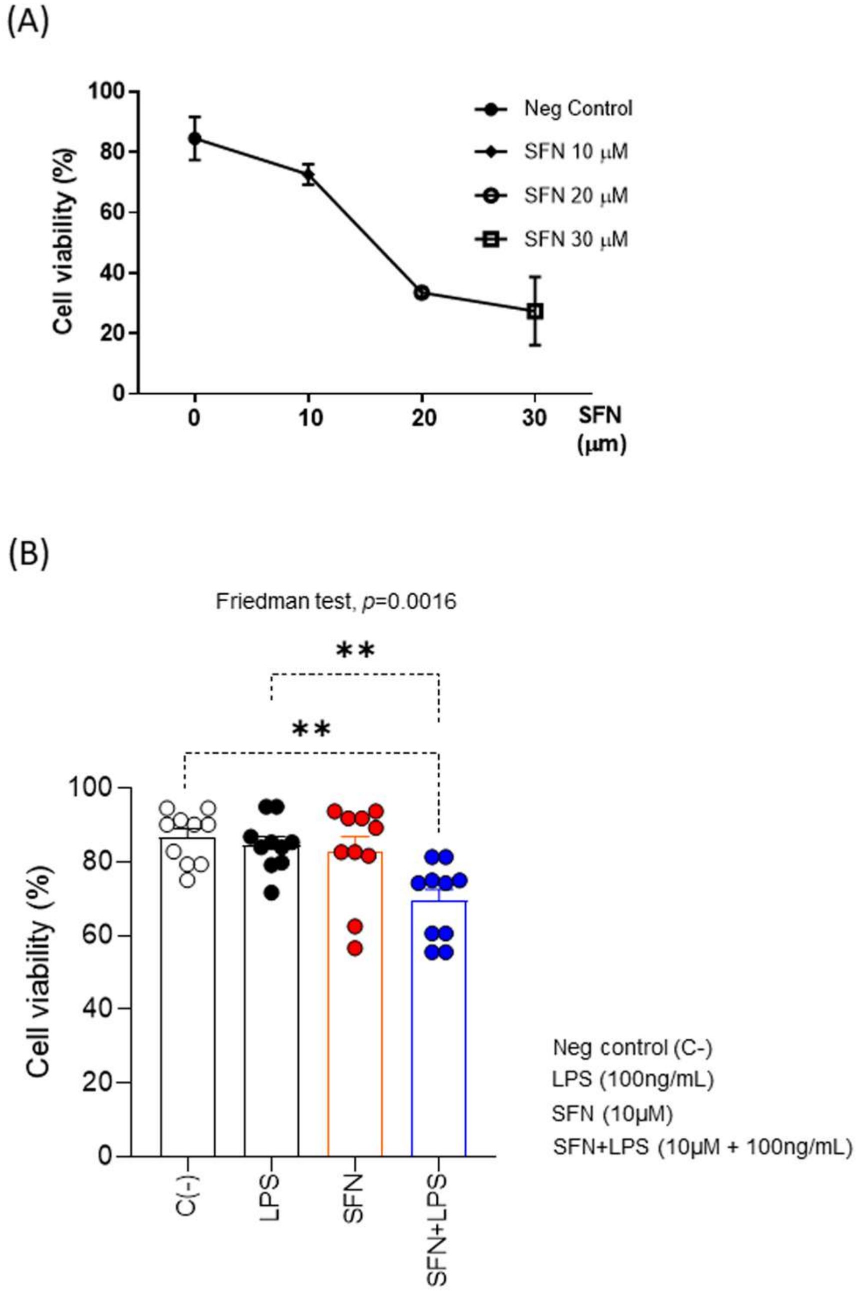

Figure S1. (A) Viability of moDCs treated with different concentrations of SFN after 48 h. Each symbol represents different SFN concentrations. (B) Cell viability percentages on moDCs under different experimental conditions. LPS: lipopolysaccharide (100 ng/mL); SFN: sulforaphane (10 μM). The bars with different symbols represent the mean and standard error of percentages of viability, necrosis and apoptosis on THP-1-cells. The Friedman test was used to detect differences in related samples across multiple comparisons, representing significant p-values. The Wilcoxon test was used for pairwise comparisons of related samples, representing significant *p*-values as * ($p<0.05$) and ** ($p<0.01$).

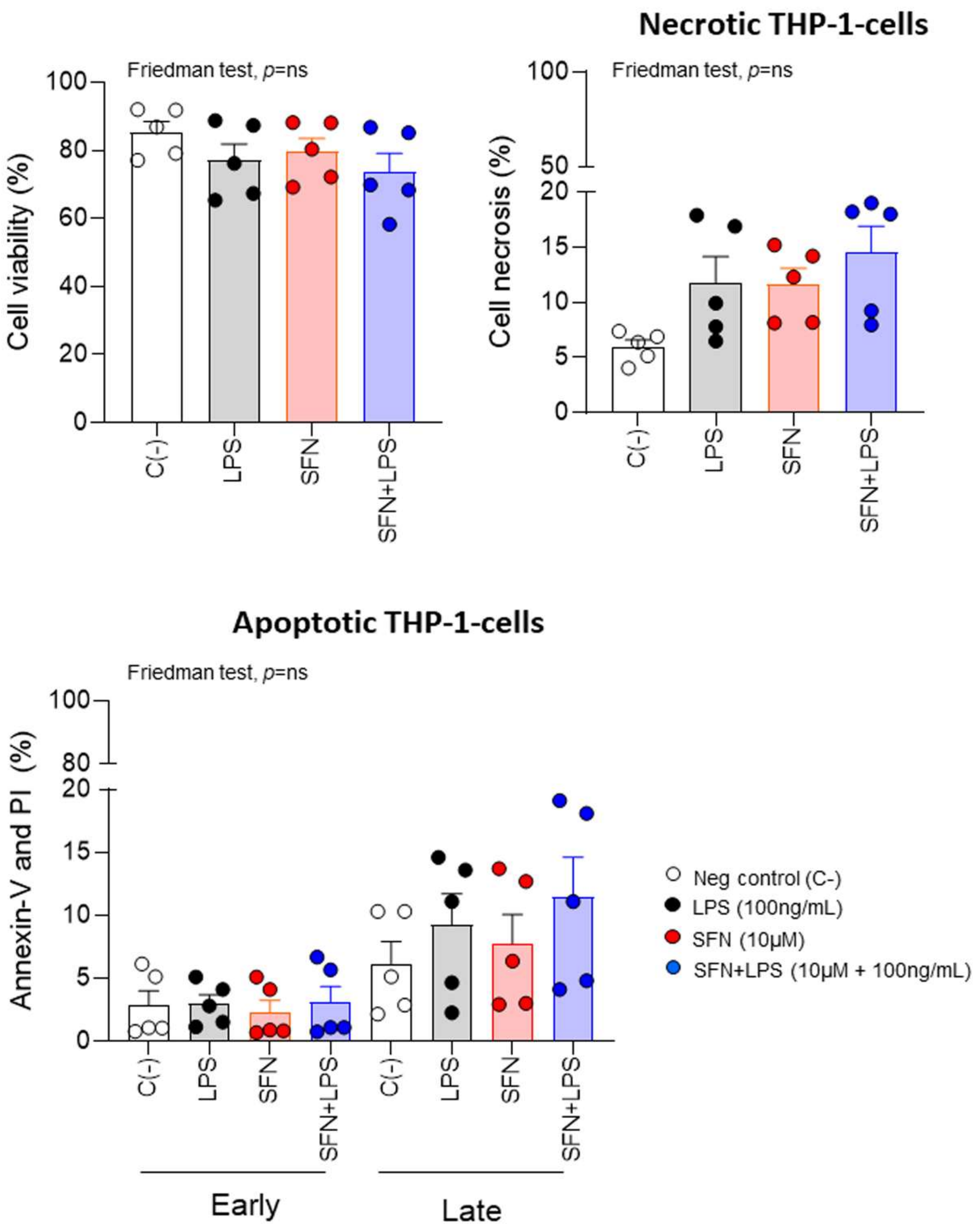

Figure S2. (Up) Viability and necrosis percentage on THP-1-cells. **(Down)** Apoptotic percentages of THP-1-cells (N = 5) under different experimental conditions. LPS: lipopolysaccharide (100 ng/mL); SFN: sulforaphane (10 μM). The bars with symbols represent the means and standard errors of percentage of viability, necrosis and apoptosis on THP-1-cells. The Friedman test was used to detect differences in related samples across multiple comparisons, representing significant *p*-values. The Wilcoxon test for pairwise comparisons in related samples, representing significant p-values as * ($p<0.05$) and ** ($p<0.01$).
